# Localization of Annexin A6 in Matrix Vesicles During Physiological Mineralization

**DOI:** 10.3390/ijms21041367

**Published:** 2020-02-18

**Authors:** Ekeveliny Amabile Veschi, Maytê Bolean, Agnieszka Strzelecka-Kiliszek, Joanna Bandorowicz-Pikula, Slawomir Pikula, Thierry Granjon, Saida Mebarek, David Magne, Ana Paula Ramos, Nicola Rosato, José Luis Millán, Rene Buchet, Massimo Bottini, Pietro Ciancaglini

**Affiliations:** 1 Departamento de Química, Faculdade de Filosofia, Ciências e Letras de Ribeirão Preto da Universidade de São Paulo (FFCLRP-USP), Ribeirão Preto, São Paulo 14040-900, Brazil; 2 Nencki Institute of Experimental Biology, 3 Pasteur Street, 02-093 Warsaw, Poland; 3 Universite Lyon 1, UFR Chimie Biochimie, CEDEX, 69 622 Villeurbanne, France; 4 ICBMS UMR 5246 CNRS, CEDEX, 69 622 Villeurbanne, France; 5 INSA, Lyon, CEDEX, 69 622 Villeurbanne, France; 6 CPE, Lyon, CEDEX, 69 622 Villeurbanne, France; 7 Université de Lyon, CEDEX, 69 622 Villeurbanne, France; 8 Department of Experimental Medicine, University of Rome Tor Vergata, 00133 Rome, Italy; 9 Sanford Burnham Prebys Medical Discovery Institute, La Jolla, CA 92037, USA

**Keywords:** Annexin A6, matrix vesicles, Langmuir monolayers, proteoliposomes, biomineralization, differential scanning calorimetry.

## Abstract

Annexin A6 (AnxA6) is the largest member of the annexin family of proteins present in matrix vesicles (MVs). MVs are a special class of extracellular vesicles that serve as a nucleation site during cartilage, bone, and mantle dentin mineralization. In this study, we assessed the localization of AnxA6 in the MV membrane bilayer using native MVs and MV biomimetics. Biochemical analyses revealed that AnxA6 in MVs can be divided into three distinct groups. The first group corresponds to Ca^2+^-bound AnxA6 interacting with the inner leaflet of the MV membrane. The second group corresponds to AnxA6 localized on the surface of the outer leaflet. The third group corresponds to AnxA6 inserted in the membrane’s hydrophobic bilayer and co-localized with cholesterol (Chol). Using monolayers and proteoliposomes composed of either dipalmitoylphosphatidylcholine (DPPC) to mimic the outer leaflet of the MV membrane bilayer or a 9:1 DPPC:dipalmitoylphosphatidylserine (DPPS) mixture to mimic the inner leaflet, with and without Ca^2+^, we confirmed that, in agreement with the biochemical data, AnxA6 interacted differently with the MV membrane. Thermodynamic analyses based on the measurement of surface pressure exclusion (π*_exc_*), enthalpy (ΔH), and phase transition cooperativity (Δt_1/2_) showed that AnxA6 interacted with DPPC and 9:1 DPPC:DPPS systems and that this interaction increased in the presence of Chol. The selective recruitment of AnxA6 by Chol was observed in MVs as probed by the addition of methyl-β-cyclodextrin (MβCD). AnxA6-lipid interaction was also Ca^2+^-dependent, as evidenced by the increase in π*_exc_* in negatively charged 9:1 DPPC:DPPS monolayers and the decrease in ΔH in 9:1 DPPC:DPPS proteoliposomes caused by the addition of AnxA6 in the presence of Ca^2+^ compared to DPPC zwitterionic bilayers. The interaction of AnxA6 with DPPC and 9:1 DPPC:DPPS systems was distinct even in the absence of Ca^2+^ as observed by the larger change in Δt_1/2_ in 9:1 DPPC:DPPS vesicles as compared to DPPC vesicles. Protrusions on the surface of DPPC proteoliposomes observed by atomic force microscopy suggested that oligomeric AnxA6 interacted with the vesicle membrane. Further work is needed to delineate possible functions of AnxA6 at its different localizations and ways of interaction with lipids.

## 1. Introduction

Bone mineralization is a tightly regulated process that involves mesenchymal cells condensing into tissue elements followed by their differentiation into cartilage (chondrocytes) or bone (osteoblasts) cells [1]. During long bone formation, occurring throughout endochondral ossification, chondrocytes mineralize their extracellular matrix (ECM) by forming apatite. Osteoblasts are responsible for the formation of bone in both endochondral and endomembranous ossification acting in concert with osteoclasts to maintain the integrity of bone tissues. Both chondrocytes and osteoblasts produce a special class of extracellular vesicles, named matrix vesicles (MVs), that provide a suitable microenvironment for initiating apatite formation during bone mineralization [2,3,4,5,6,7,8]. MV-mediated mineralization is orchestrated by a constellation of proteins. Among them, the most abundant proteins in MVs isolated from hypertrophic chondrocytes [9] and human osteoblast-like cells Saos2 [10] are the annexins, AnxA1, AnxA2, AnxA5, AnxA6, and AnxA7. 

Annexins belong to a family of structurally related proteins which bind to negatively charged phospholipids in a Ca^2+^-dependent manner. However, their association with membranes can be also Ca^2+^-insensitive [11,12]. The interaction between annexins and negatively charged phospholipids is also dependent on the presence of cholesterol (Chol) [11] and pH [13,14]. Annexins also interact with collagen: AnxA5- and AnxA6-modified proteoliposomes bind to type II collagen [15,16,17], while AnxA6 in MVs binds to type I collagen [18]. AnxA6 is the largest member of the annexin family present in MVs, with a molecular weight (MW) of approximately 68 kDa [19]. Unlike other members of the annexin family, which are structurally characterized by the presence of a highly conserved core composed of four homologous domains, AnxA6 has eight domains [19]. The differences between AnxA6 and the other members of the annexin family are also functional, especially in regard to membrane folding [20]. So far, little is known about the localization of annexins in MVs and their interaction with the MV membrane. Herein, we investigated the localization of AnxA6 in chicken embryo cartilage MVs, using a freeze-thaw procedure with and without ethylene glycol tetraacetic acid (EGTA). The co-localization of AnxA6 with Chol domains in MVs was probed by the addition of methyl-β-cyclodextrin (MβCD). To determine the nature of the interaction between AnxA6 and lipids, monolayers and bilayers of different lipid compositions containing AnxA6 were analyzed by means of exclusion pressure analysis, differential scanning calorimetry (DSC), dynamic light scattering (DLS), and atomic force microscopy (AFM). Our findings provide insights into a possible process of AnxA6 translocation across the MV membrane during biomineralization and we propose that AnxA6 has distinct functions in each translocation step from the lumen (interior) to the exterior surface of the MV membrane during biomineralization. 

## 2. Results and Discussion 

### 2.1. AnxA6 Associates With the MV Membrane as Both an Integral and a Peripheric Protein

**Figure 1:**
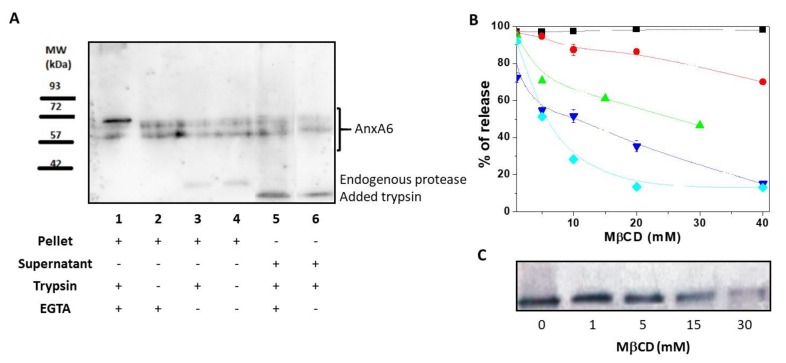
Annexin A6 in matrix vesicles. (**A**) Western blotting performed using mouse anti-AnxA6 monoclonal antibody to detect two isoforms of annexin A6 (AnxA6) in matrix vesicles (MVs) in the presence or absence of 0.1% trypsin after four freeze-thaw procedures followed by centrifugation. Lanes 1, 2, 3, and 4 corresponded to pellets, while lanes 5 and 6 corresponded to supernatants. MVs were treated with or without trypsin, with 2 mM Ca^2+^ or with 2 mM ethylene glycol tetraacetic acid (EGTA), as indicated. (**B**) AnxA6 co-localizes with cholesterol. MVs were incubated in synthetic cartilage lymph (SCL) medium containing 10 mM EGTA and an increasing concentration of methyl-β-cyclodextrin (MβCD) for 30 min in room temperature. Then, the samples were centrifuged. Lactate dehydrogenase (▼) and tissue non-specific alkaline phosphatase (TNAP) (●) activities were tested in the pellets and in the supernatants, as described in Materials and Methods. In the pellets, only the content of cholesterol (◆) and protein (■) was measured. MβCD-treated pellets were analyzed to determine AnxA6 (▲) content by Western blotting using a rabbit polyclonal anti-AnxA6 antibody that recognizes a single protein band. (**C**) Increasing MβCD concentrations led to a decrease of AnxA6 content. The results are expressed as a percentage of release of MV components ± SD.

First, we assessed the association of AnxA6 with the MV membrane by using a freeze-thaw procedure [21]. After 4 freeze-thaw cycles of MV solutions, the presence of full-length transmembrane AnxA6 embedded within the MV bilayer was evidenced by the presence of two isoforms of AnxA6 with a MW of approximately 67 and 60 kDa, respectively, into the pellet. These isoforms were insensitive to trypsin treatment and were not released into the supernatant by addition of EGTA (Figure 1A, lanes 1 and 2). Another population of full-length AnxA6 interacted strongly with the membrane in a Ca^2+^-dependent manner as indicated by the appearance of an additional small AnxA6 fragment with a MW of approximately 35 kDa. The presence of a 35 kDa-MW AnxA6 fragment in MVs has been previously reported [22] and is due to the collagenase treatment and/or action of endogenous proteases [23,24]. It indicates that a population of AnxA6 associates with the inner leaflet of the MV membrane, accessible to endogenous proteases, as confirmed by the treatment with (Figure 1A, lane 3) or without (Figure 1A, lane 4) trypsin that did not affect the intensity of the bands. 

A part of AnxA6 was digested by trypsin as indicated by the appearance of a 30 kDa-MW band (Figure 1A, lanes 5 and 6). Addition of EGTA (Figure 1A, lane 5) or 2 mM Ca^2+^ (Figure 1A, lane 6) did not eliminate the 30 kDa-MW band. The fragment was released by the freeze-thaw procedure in the supernatant, suggesting that it associates with the outer leaflet of the MV membrane due to its accessibility to trypsin digestion. The two full-length isoforms that were released in the supernatant could originate from the soluble AnxA6 inside MVs (Figure 1A, lane 5) or EGTA-released AnxA6 interacting with the inner leaflet (Figure 1A, lane 6) since both of them were not subjected to trypsin digestion. 

### 2.2. AnxA6 Co-Localizes in Lipid Domains Enriched in Cholesterol

The MV membrane is enriched in Chol [25], therefore, we assessed if AnxA6 co-localizes in Chol-enriched lipid domains by examining the effect of the Chol-depleting agent methyl-β-cyclodextrin (MβCD) on AnxA6 release from the MV membrane. MβCD released Chol from the MV membrane in a concentration-dependent manner, reaching a maximal efficiency of release at a concentration of 15–20 mM (Figure 1B). The remaining 15% percent of membrane Chol, which was resistant to MβCD treatment, represented Chol esters. The calculated molar ratio between free Chol and Chol esters was 7.4 ± 1.0 mol/mol. Approximately 20% of glycosylphosphatidylinositol (GPI)-linked tissue non-specific alkaline phosphatase (TNAP), as probed by its activity, was released from the MV membrane at the maximal MβCD concentration. In addition, the activity of lactate dehydrogenase (LDH), a cytosolic marker, served to monitor the integrity of the MV membrane. Addition of MβCD affected significantly the integrity of the MV membrane at concentrations greater than 1 mM (Figure 1B) as indicated by the decreasing LDH activity in the supernatant. However, the total protein content remained almost stable (it decreased only slightly) in the pellet during the treatment with MβCD at concentrations ranging from 0 mM to 40 mM. Conversely, a significant release of AnxA6 from MV pellets into the supernatant was observed already when 15 mM MβCD was added to the medium (Figure 1B,C). This finding suggested that AnxA6 co-localizes in lipid domains enriched in Chol and its release is selective since the total protein concentration in the pellet did not change significantly (Figure 1B). 

### 2.3. Interaction of AnxA6 With Lipid Monolayers

**Figure 2:**
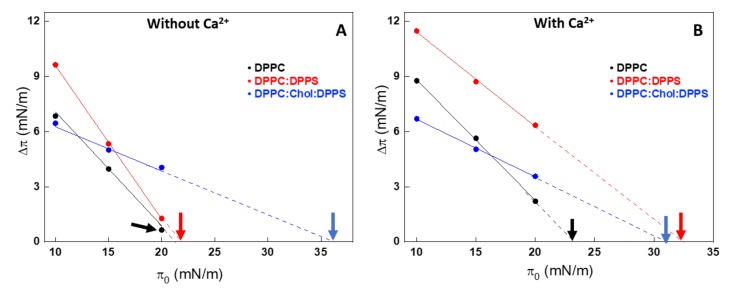
Changes in the surface pressure (Δπ) due to the injection of AnxA6 as a function of the initial surface pressure (π_0_) of Langmuir monolayers in the absence (**A**) and presence of 2 mM Ca^2+^ (**B**) for 1,2-dipalmitoyl-*sn*-glycero-3-phosphocholine (DPPC) (black dots), 9:1 DPPC:1,2-dipalmitoyl-*sn*-glycero-3-phospho-L-serine (DPPS) (red dots) and 5:4:1 DPPC:cholesterol (Chol):DPPS (blue dots) monolayers as a consequence of interaction with AnxA6. The intercept with abscissa is the exclusion pressure (π*_exc_*) and is indicated by the arrows. The analyses were adjusted to π_0_ close to 10, 15, and 20 mN/m.

In order to assess how AnxA6 interacts with the leaflets of the MV membrane bilayer, we used Langmuir monolayers formed on the surface of a pendant drop. A monolayer made of 1,2-dipalmitoyl-*sn*-glycero-3-phosphocholine (DPPC) was used to mimic the outer leaflet, whereas the inner leaflet was modeled with a 9:1 DPPC:1,2-dipalmitoyl-*sn*-glycero-3-phospho-L-serine (DPPS) monolayer. Saturated lipids were chosen due to their relative high amount in MVs (approximately 48% are saturated fatty acids) [26]. The effect of Chol was monitored by comparing these monolayers with a 5:4:1 DPPC:Chol:DPPS monolayer. The interaction of AnxA6 with the monolayers was assessed by measuring the value of exclusion pressure (π*_exc_*). The linear dependency between Δπ and π_0_ for all the monolayers, both in presence and absence of 2 mM Ca^2+^, enabled us to obtain the abscissa intercept corresponding to π_exc_ (arrows in Figure 2). 

Higher π_exc_ values indicate a higher interaction or penetration of the protein into the monolayer even at higher packing conditions. Addition of Chol induced an increase in π_exc_ for all the lipid monolayers tested, indicating a strong interaction between the protein and the mixed monolayers in the presence of this sterol. This behavior was well observed in Chol-containing lipid monolayers, both in the absence (Figure 2A) and presence (Figure 2B) of Ca^2+^. The presence of Ca^2+^ significantly contributed to the increased interaction of AnxA6 with the DPPS-containing monolayer, 9:1 DPPC:DPPS (molar ratio), as revealed by the increase in π_exc_ values from 23.7 mN/m in the absence of Ca^2+^ to 32.9 mN/m in the presence of Ca^2+^ (Table 1). 

### 2.4. Interaction of AnxA6 With Lipid Bilayers 

The interaction of AnxA6 with liposomes was assessed using the same lipid compositions of the Langmuir monolayers. In general, the addition of Ca^2+^ led to a significant increase in protein association with all the lipid bilayers (Table 1). It is worth noting that addition of Ca^2+^ led to a significant increase in the amount of AnxA6 incorporated into 5:4:1 DPPC:Chol:DPPS liposomes. However, it did not lead to a significant change in π_exc_. This apparent controversial result can be assigned to the protein penetration into the monolayers in the presence and absence of Ca^2+^. However, the stronger interaction with the lipids in the presence of Ca^2+^ translated into a greater incorporation into the liposomes.

**Figure 3:**
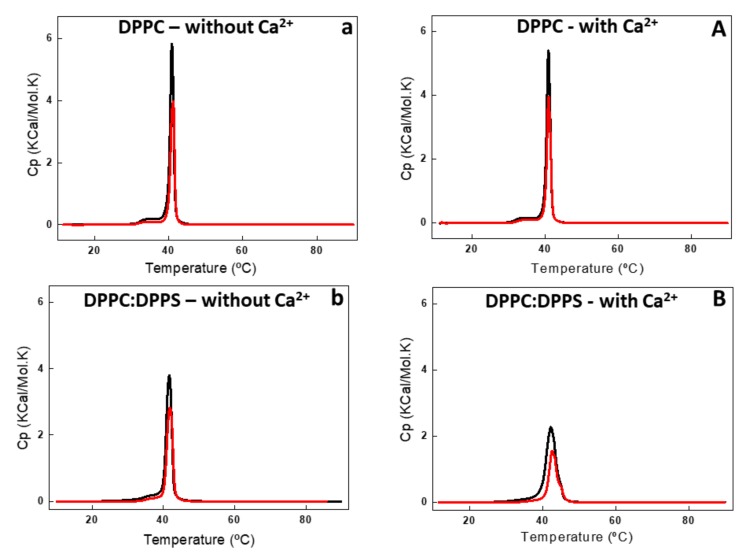
Differential scanning calorimetry (DSC) thermograms of DPPC (**a**,**A**) and 9:1 DPPC:DPPS (**b**,**B**) liposomes (10 mg/mL, total lipid concentration) in the absence (lower case letter) and in the presence (capital letter) of 2 mM Ca^2+^. DSC thermograms were processed in excess heat capacity (C_p_) (kcal/K.mol) as function of temperature (°C) of liposomes (black line) and proteoliposome (red line) harboring AnxA6.

**Figure 4:**
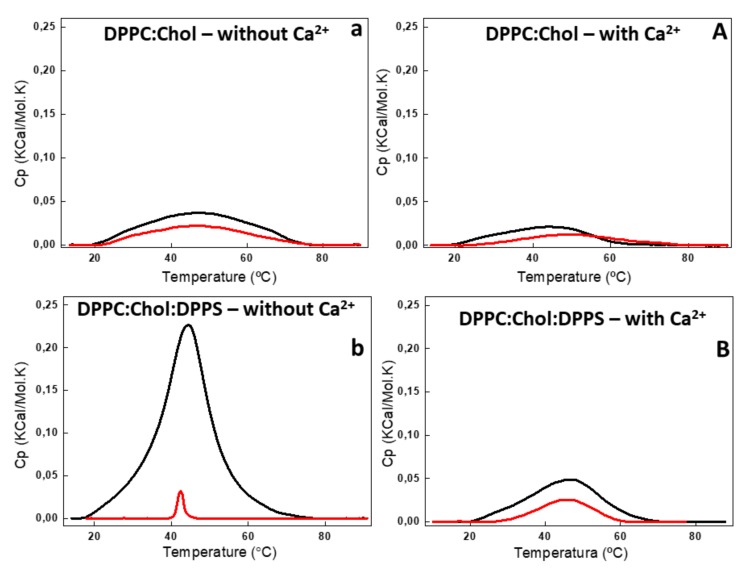
DSC thermograms of 6:4 DPPC:Chol (**a**,**A**) and 5:4:1 DPPC:Chol:DPPS (**b**,**B**) liposomes (10 mg/mL, total lipid concentration) in the absence (lower case) and in the presence (capital letter) of 2 mM Ca^2+^. DSC thermograms were processed in excess heat capacity (C_p_) (kcal/K.mol) as function of temperature (°C) of liposomes (black) and AnxA6-harboring proteoliposome (red).

DSC analysis showed the effect of AnxA6 addition on the thermodynamic behavior of liposomes (Figure 3 and Figure 4, and Table 2). Addition of AnxA6 decreased the value of enthalpy (ΔH) for all lipid membranes irrespective of the presence of Ca^2+^ (Table 2). For DPPC vesicles, there were also no significant changes in the values of phase transition cooperativity (Δt_1/2_) and transition phase temperature (T_m_) (Figure 3a,A). 

The addition of AnxA6 led to a decrease in the Δt_1/2_ values in the presence of Ca^2+^ from 2.80 to 2.29 for 9:1 DPPC:DPPS proteoliposomes containing negative charges (Figure 3b,B) and from 19.37 to 13.89 for 5:4:1 DPPC:Chol:DPPS proteoliposomes (Table 2). However, the presence of Chol increased strongly the Δt_1/2_ values irrespective of the presence of Ca^2+^, resulting in much more cooperative phase transitions and displaying the synergistic effect of protein, Ca^2+^, negative charges, and Chol, compared to Chol-devoid liposomes (Figure 4a,A,b,B). 

The changes in the lipid bilayers’ thermodynamic parameters caused by Chol were concentration dependent [27,28,29]. Addition of 40% molar percentage of Chol to the phospholipids eliminated the pretransition phase of DPPC bilayers (±35 °C) and also resulted in a broadened main lipid phase transition peak, leading to a decrease of ∆H and increase of Δt_1/2_ values (Table 2). The ternary 5:4:1 DPPC:Chol:DPPS composition, as well as the binary 6:4 DPPC:Chol composition, showed a significant decrease in ∆H in the presence and absence of Ca^2+^ (Figure 4) as compared with Chol-devoid liposomes (Figure 3). The addition of AnxA6 decreased the value of ∆H of the lipid phase transition in both the ternary 5:4:1 DPPC:Chol:DPPS and binary 6:4 DPPC:Chol compositions. This effect was Ca^2+^-dependent for both the systems.

The largest ΔH decrease upon addition of AnxA6 was observed for the binary 9:1 DPPC:DPPS composition in the presence of Ca^2+^ (from 8.72 to 4.96 Kcal/mol) and for the ternary 5:4:1 DPPC:Chol:DPPS composition in the absence of Ca^2+^ (from 1.14 to 0.43 Kcal/mol) (Table 2, Figure 3B and Figure 4a). Such a decrease is consistent with AnxA6 interacting with the surfaces of the bilayer or being incorporated into the bilayer, preventing lipid molecules from participating in the phase transition. Such changes in the thermodynamic parameters should be taken into account and correlated to the interactions of the protein with the phospholipids and, consequently, with the physical stability of the liposome formulation.

Taken together, the calorimetry studies strongly indicate that AnxA6 can also associate with the outer leaflet of the MV membrane regardless of the presence of phosphatidylserine (PS), sterol, or calcium. However, the best results for protein interaction with liposomes were achieved in more fluid vesicles containing negative charges, Chol, and calcium. It is possible that AnxA6 exhibits different types of membrane interaction depending on the lipid composition, since different changes in the thermodynamic parameters were obtained depending on the proteoliposomes’ lipid composition. These results corroborate the hypothesis that AnxA6 can be found in distinct regions of the MV membrane.

Finally, DLS confirmed that the diameter of the liposomes was smaller than 200 nm in absence of Ca^2+^ and did not show significant changes upon addition of Ca^2+^ to vesicle solution. The polydispersity index (PI) was smaller than 0.02 for all the formulations. The vesicles were stable in size (within +/-10 nm) for 28 days as probed by DLS.

### 2.5. Atomic Force Microscopy Analysis

**Figure 5:**
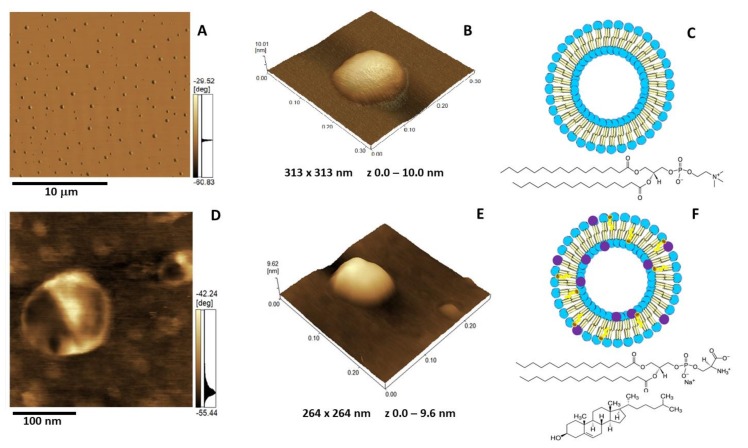
Atomic force microscopy (AFM) images of DPPC (**A**–**C**) and 5:4:1 DPPC:Chol:DPPS (**D**–**E**) liposomes (1.5 mg/mL): (**A**,**D**) Phase image, (**B**,**E**) 3D topography, and (**C**,**F**) liposome schematic representation. The blue and purple circles in **C** and **F** are assigned to the polar heads of DPPC and DPPS, respectively. The brown and yellow colors are assigned to Chol. The chemical structure of DPPC is depicted in **C**, and the chemical structures of DPPS and Chol are depicted in **F**.

**Figure 6:**
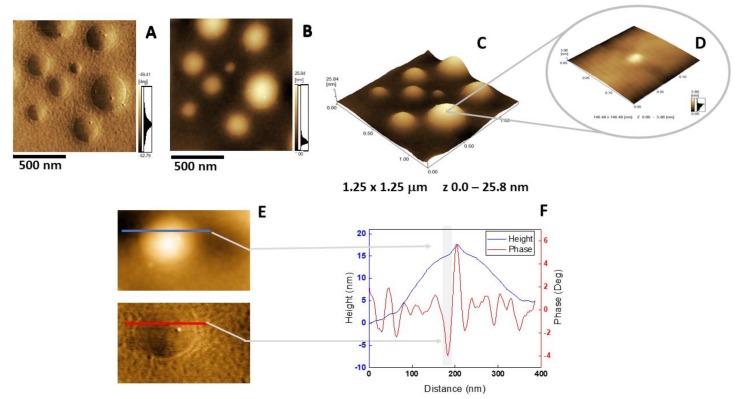
AFM analysis of DPPC proteoliposomes harboring AnxA6: (**A**) Phase image, (**B**) height image, (**C**) 3D topography, and (**D**) magnification of a 146.48 × 146.48 nm^2^ surface area showing a protein domain; (**E** and **F**) phase and height line analysis (blue and red lines) of an AnxA6 domain inserted in DPPC proteoliposomes (the gray shadow highlights the protrusion). (**E**) was prepared using WSxM 4.0 Beta 9.1 software developed by Horcas et al. [33].

**Figure 7:**
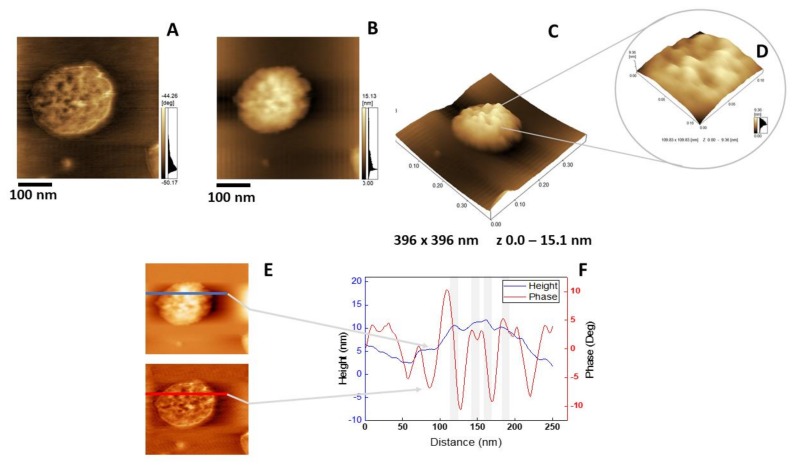
AFM analysis of 6:4:1 DPPC:Chol:DPPS proteoliposomes harboring AnxA6: (**A**) Phase image, (**B**) height image, (**C**) 3D topographic profile, and (**D**) magnification of a 109.83 × 109.83 nm^2^ surface area showing protein domains; (**E** and **F**) phase and height line analysis (blue and red lines) of an AnxA6 domain inserted into 6:4:1 DPPC:Chol:DPPS proteoliposomes (the gray shadow highlights the protrusions). (**E**) was prepared using WSxM 4.0 Beta 9.1 software developed by Horcas et al. [33].

The insertion of AnxA6 into lipid bilayers was further validated by atomic force microscopy (AFM) topographic and phase imaging. Liposomes composed of DPPC (Figure 5A–C) and DPPC:Chol:DPPS (6:4:1) (Figure 5D–F) showed spherical particles with a uniform distribution of size and a smooth and homogeneous surface without significant changes in the phase (Figure 5A,D). Conversely, AnxA6-harboring proteoliposomes showed surface irregularities formed by protrusions that appear to agglutinate at various sites (Figure 6 and Figure 7). By recording the AFM topographic cross-sections, we found that these protrusions were 21.37 ± 4.83 nm wide and 1.15 ± 0.41 nm tall (N = 20) for DPPC proteoliposomes (Figure 6), whereas they were 26.97 ± 7.62 nm wide and 2.01 ± 0.89 nm tall (N = 22) for 6:4:1 DPPC:Chol:DPPS proteoliposomes (Figure 7). The protrusions on the surface of vesicles observed by AFM imaging suggested that oligomeric AnxA6 was on the surface of DPPC proteoliposomes (Figure 6). AnxA6 has two flexible lobes [30], thus it may adopt several conformations [31] with the lobes binding the monolayer in either a parallel orientation or an antiparallel orientation or with one of the lobes protruding out from the monolayer [32].

## 3. Materials and Methods

### 3.1. Materials

All aqueous solutions were prepared using Millipore^®^ DirectQ ultrapure water. Bovine serum albumin (BSA), tris hydroxymethyl-amino-methane (Tris), sodium dodecylsulfate (SDS), and glutaraldehyde (Grade I, purified for use as an electron microscopy fixative) were obtained from Sigma Aldrich (Sigma Aldrich Corp., St. Louis, MO, USA). Bradford reagent was from Bio-Rad, (Bio-Rad Laboratories, Inc., Hercules, CA, USA). DPPC and Chol were purchased from Avanti Polar Lipids (Avanti Polar Lipids, Inc., Alabaster, AL, USA), whereas DPPS was obtained from Sigma Aldrich. All reagents were of analytical grade and used without further purification.

### 3.2. Expression of AnxA6 

Recombinant human AnxA6 protein was expressed and purified as described by Bandorowicz-Pikula et al. [34].

### 3.3. Isolation of Matrix Vesicles 

MVs were isolated from 17–day-old chicken embryos, as described before [3], excluding the trypsin-digestion step. Briefly, leg bones from 20 animals were isolated, cut into 1–3-mm-thick slices and extensively washed in ice cold synthetic cartilage lymph (SCL) containing 1.42 mM Na_2_HPO_4_, 1.83 mM NaHCO_2_, 12.7 mM KCl, 0.57 mM MgCl_2_, 100 mM NaCl, 0.57 mM Na_2_SO_4_, 5.55 mM glucose, 63.5 mM sucrose, and 16.5 mM 2-[(2-hydroxy-1,1-bis(hydroxymethyl)ethyl)amino]-ethanesulfonic acid (TES), pH 7.4. The bones were digested with collagenase type I from Clostridium histolyticum (200 U of collagenase/g of tissue in SCL medium containing 1 mM calcium) at 37 °C for 3 h in SCL. One µg/mL of protease inhibitor cocktail (Sigma Aldrich) was added and the digested tissue was filtrated and centrifuged at 800× *g* for 30 min at 4 °C. The pellet was discarded, and the supernatant was centrifuged at 30,000× *g* for 30 min at 4 °C. The second supernatant was subjected to a 250,000× *g* centrifugation for 30 min and the supernatant was discarded, whereas the pellet was washed three times in SCL medium without Ca^2+^ to remove calcium and protease inhibitor. The final volume of the MV solution was 400 µL and stored at 4 °C for further analysis. 

### 3.4. Enzyme Activities, Total Protein, and Cholesterol Content 

The activity of TNAP in MVs was determined by adding 10 mM *p*-nitrophenyl phosphate in 0.56 M amino 2-methyl 2-propanol and 1mM MgCl_2_ buffer, pH 10.4, to the MV solution [35]. The appearance of the product (*p*-nitrophenolate) was monitored at 37 °C by measuring the absorbance at 405 nm. For standardization, the total protein content was measured in 2 µL of MV suspension supplemented with 798 µL of pure water and 200 µL of Bradford reagent (Bio-Rad). Total protein concentration was measured at 595 nm. Bovine serum albumin served as protein standard. TNAP (specific) activity was 8–10 U/mg and protein concentration was 1–2 mg/mL. To measure the activity of LDH, aliquots of MV solution were mixed with an equal volume of 0.5% Triton X-100 in order to completely release the enzyme from the vesicles. The reaction mixture contained 1 mM pyruvate and 0.25 mM nicotinamide adenine dinucleotide reduced (NADH). The oxidation of NADH was determined by measuring the absorbance at 340 nm [36]. The content of Chol in MVs after treatment with methyl-β-cyclodextrin (MβCD) was measured by Amplex Red Cholesterol Assay (Thermo Fisher Scientific, Waltham, MA, USA) according to the manufacturer’s instruction. The measurement was performed without or with Chol esterase to determine free Chol (FC) to Chol esters (CE) (mol/mol) ratio. The ratio was calculated as follows: FC/CE = (F_FC_)/(F_T_-F_FC_) where F_T_ and F_FC_ are the fluorescence of resorufin measured at 584 nm in the presence (total Chol) and absence (free Chol) of Chol esterase, respectively.

### 3.5. Treatment of Matrix Vesicles With Trypsin and Freeze-Thaw Cycles in the Absence and Presence of Calcium

The MV solution was divided in four parts. The samples were mixed with 2 mM CaCl_2_ without and with 0.1% trypsin and 2 mM EGTA without and with 0.1% trypsin, respectively. Trypsin served to monitor AnxA6 fragments by indicating AnxA6 accessible to protease digestion. Each sample was incubated at room temperature for 1 h, then it was centrifuged at 170,000× *g* for 20 min at room temperature. The supernatants were discarded, whereas the pellets were supplemented with 150 μL of SCL medium and subjected to four freeze-thaw cycles. Each cycle consisted of sample incubation in liquid nitrogen for 5 min and thawing at 37 °C for 5 min followed by vortexing for 1 min. The freeze-thaw cycles were used to disrupt and re-fuse the vesicles, during which time the solute equilibrates between the inside and outside [21]. Each sample was centrifuged to release AnxA6 in the supernatant and the membranous fraction of AnxA6 in the pellet. 

### 3.6. Western Blotting 

Proteins were separated by SDS-polyacrylamide gel electrophoresis (SDS-PAGE) on 10% gels [37] and transferred onto nitrocellulose membrane (GE Healthcare Bio-Sciences Corp., Pittsburgh, PA, USA). The membrane was blocked with 5% low-fat milk solution in 20 mM Tris, pH 7.5, and 500 mM NaCl for 60 min at room temperature, washed, and then incubated with mouse anti-AnxA6 monoclonal antibody (1:1000 v/v; BD Biosciences, San Jose, CA; this antibody recognizes two AnxA6 isoforms) or rabbit anti-AnxA6 polyclonal antibody (1:5000 v/v; Abcam, Cambridge, MA, USA; this antibody is nonspecific to AnxA6 isoforms) in 3% low-fat milk, 0.05% Tween-20, 20 mM Tris, pH 7.5, and 500 mM NaCl overnight at 4 °C. The membrane was washed in the same buffer and incubated for 60 min with anti-mouse immunoglobulin (IgG) conjugated with horseradish peroxidase (GE Healthcare Bio-Sciences). The bands were visualized using enhanced chemiluminescence (ECL) Western Blotting Detection Reagents (GE Healthcare Bio-Sciences) according to the manufacturer’s instruction and exposing nitrocellulose to Hyperfilm ECL (GE Healthcare Bio-Sciences). 

### 3.7. Treatment of Matrix Vesicles with Methyl-β-Cyclodextrin

MVs at a protein concentration of 0.33 mg/mL were incubated with increasing concentrations of methyl-β-cyclodextrin (MβCD) in SCL containing 10 mM EGTA. After 30 min of incubation at room temperature, the samples were centrifuged at 170,000× *g* for 20 min at room temperature. The supernatants were stored at −20 °C for enzyme activity assays, whereas the pellets were washed 3 times with SCL containing 10 mM EGTA and resuspended in 50 μL of the same buffer. One-half of each pellet was utilized to assess the presence of AnxA6, whereas the other half was used to measure TNAP and LDH activities as well as the total protein and Chol concentrations.

### 3.8. Preparation of Liposomes

DPPC, DPPC:DPPS (9:1), DPPC:Chol:DPPS (5:4:1), and DPPC:Chol (6:4) (molar ratios) liposomes (referred to as 9:1 DPPC:DPPS, 5:4:1 DPPC:Chol:DPPS, and 6:4 DPPC:Chol, respectively) were prepared as previously described using a 50 mM Tris-HCl buffer, pH 7.5, containing 2 mM MgCl_2_ to yield a final solution with 10 mg/mL of lipids [38,39,40,41].

### 3.9. Preparation of Proteoliposomes

AnxA6 (0.2 mg/mL) was incorporated into liposomes by direct insertion in 50 mM Tris-HCl buffer, pH 7.5, containing 2 mM MgCl_2_ with or without 2 mM CaCl_2_ in a 1:100 protein:lipid ratio. The mixture was incubated for 24 h at 25 °C and ultracentrifuged at 100,000× *g* for 1 h, at 4 °C. The pellet containing proteoliposomes was suspended to the original volume in the same buffer [42]. Protein concentration was measured in presence of 0.2 g/mL SDS [43]. Bovine serum albumin (BSA) was used as a standard.

### 3.10. Dynamic Light Scattering (DLS)

Liposomes and proteoliposomes were filtered through 0.8-μm pore size membranes (Merck Millipore, Burlington, MA), dispersed in deionized water [38,39,40,41], and the size distribution and polydispersity index (PI) were determined by DLS using an N5 Submicron Particle Size Analyzer (25mW Helium-Neon Laser-632.8 nm, 90°) (Beckman Coulter, Fullerton, CA). The average value (N = 5) of the vesicles’ diameter was obtained at 25 °C by unimodal distribution. 

### 3.11. Differential Scanning Calorimetry (DSC)

Transition phase temperature (T_m_), enthalpy (ΔH), and phase transition cooperativity (Δt_1/2_) of large unilamellar vesicles (LUVs) prepared with different lipid compositions (as described in Section 3.8) were assessed by DSC. LUVs suspensions and reference buffer employed in the experiment were previously degasified under vacuum (140 mbar) for 30 min. The samples and reference (buffer) were scanned from 10 to 90 °C at an average heating rate of 0.5 °C/min, under pressure of 3 atmosphere (atm), and the recorded thermograms were analyzed by means of a Nano-DSC II (Calorimetry Sciences Corporation (CSC), Lindon, UT). The baseline was determined by filling the sample and reference cells with buffer solution. The data were analyzed using the software package CpCalc provided by CSC. The thermograms shown in the figures correspond to the first scan, but at least three heating-cooling cycles were performed for each analysis and ±5% variation [40].

### 3.12. Exclusion Pressure (π_exc_)

The incorporation of AnxA6 in preformed monolayers was monitored using a pendent drop system as described by Andrade et al. [44]. The pendent drop (approximately 11–12 µL) consisted of a microenvironment formed by 50 mM Tris-HCl buffer, pH 7.5, containing 2 mM MgCl_2_ with or without 2 mM CaCl_2_. Initially, the monolayers were formed by injection of a few microliters of lipid solution in chloroform into the drop until the monolayer’s surface tension (γ_M_) of interest was reached. A waiting period of 5 min was applied to stabilize the monolayer. Changes in γ_M_ were monitored by the axisymmetric drop shape analysis (ADSA) technique following injection of 0.2 μL of Anx6 aqueous solution into the drop (0.25 mg/mL). The interaction of the protein with the monolayer was detected by changes in γ_M_. The surface pressure (π) was calculated by subtracting the surface tension of the water drop (γ_0_) from γ_M_ (π= γ_0_-γ_M_). The changes in surface pressure (Δπ) after the injection of AnxA6 were plotted against π_0_ and the values of π_exc_ were calculated by the extrapolation of the curve to a nil value of Δπ [44].

### 3.13. Atomic Force Microscope (AFM) Imaging 

Liposomes and proteoliposomes were filtered through 0.8-μm pore size membranes (Merck Millipore) and stabilized by adding 1:1 (v/v) glutaraldehyde (~5% final concentration) to avoid vesicle deformation and disruption. The mixtures were homogenized, and then 8 μL of the sample were dropped onto freshly cleaved mica substrates, left to dry at room temperature, and imaged by an AFM (model SPM-9600 Scanning Probe Microscope, Shimadzu Corporation, Japan) operating in tapping mode as described previously by Bolean et al. [41]. Scanning was performed in air at 25 °C by using silicon probes with a resonance frequency ranging from 324 to 369 kHz (Nanosensors™, Neuchâtel, Switzerland). The scan rate was set at 0.2–0.3 Hz to prevent tip-induced vesicle deformations and/or damages. The used cantilevers had a spring constant of a 38 ± 8 N/m and a resonance frequency of 336 ± 67 kHz. The roughness values and simultaneous analysis of phase and height were carried by using SPM Offline software (Shimadzu) and treated by using WSxM 4.0 Beta 9.1 and Origin 2019 software [33,45]. Three distinct experiments were carried out for each type of vesicles and N = 100 vesicles were analyzed in each experiment. 

## 4. Conclusions

In this study, we investigated the association of AnxA6 with the membrane bilayer of natural MVs and with MV biomimetics. By using biochemical procedures on MVs isolated from 17-day-old chicken embryos, AnxA6 was found to interact with the MV membrane as both an integral and peripheral protein, which suggests distinct lipid–protein interactions and possible functions. Of particular interest is the strong co-localization of AnxA6 with Chol. This result is coherent with the Chol-driven recruitment of AnxA6 from the lumen of the cell toward plasma membrane [46]. 

Studies based on monolayers and proteoliposomes as MV biomimetics strengthened our findings by shedding the light on the interactions of AnxA6 with different lipid systems in distinct ways. Of particular importance is that AnxA6 induced an increase in the surface pressure and a decrease in the enthalpy of both DPPC (mimicking the outer leaflet of the MV bilayer) and 9:1 DPPC:DPPS (mimicking the inner leaflet of the MV bilayer) lipid monolayers indicating an interaction of AnxA6 with these lipid systems. However, the interaction of AnxA6 with bilayers containing Chol is probably deeper since it led to a strong increase in phase transition cooperativity and increased the packing of the lipid membrane.

The Ca^2+^ dependence of lipid–protein interaction was well evidenced by the increase in surface pressure caused by the addition of AnxA6 to negatively charged lipid monolayers composed of 9:1 DPPC:DPPS in presence of Ca^2+^ with respect to neutral monolayers composed of DPPC and in absence of Ca^2+^. AnxA6 interacted differently with DPPC and 9:1 DPPC:DPPS monolayers even in the absence of Ca^2+^ as observed by the larger change in phase transition cooperativity in 9:1 DPPC:DPPS vesicles as compared to DPPC vesicles. 

AFM images showed protrusions that suggest domains of oligomeric AnxA6 on the surface of DPPC proteoliposomes, thus AnxA6 may adopt several conformations upon interaction with the lipid membrane. 

**Figure 8:**
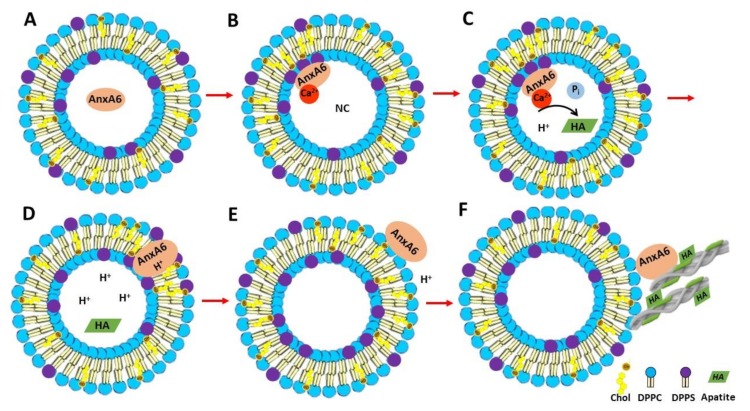
Representation of the mechanism of AnxA6 translocation during MV-mediated mineralization and its putative functions. (**A**) AnxA6 is localized in the lumen of MVs at low calcium concentrations. (**B**) Accumulation of calcium inside MVs favors the binding of AnxA6 to the inner leaflet of the MV membrane bilayer containing phosphatidylserine (PS) in a Ca^2+^-dependent manner, forming a nucleational core (NC) and initiating apatite formation. (**C**) During apatite formation, protons (H^+^) are released, inducing the protonation of AnxA6 and rendering it more hydrophobic due to an ionization potential around 5.5. (**D**) Protonated AnxA6 can translocate within the MV bilayer where it may form an ion pore. (**E**) The pH outside MVs corresponds to that of the extracellular matrix and is neutral. AnxA6 can be deprotonated and released to the external surface of MVs enriched in phosphatidylcholine (PC). (**F**) AnxA6 binding to neutral phospholipids is Ca^2+^-independent. AnxA6 may also bind to collagen fibers, contributing to the adhesion of MVs to collagen fibers.

Taken together, these findings substantiate a possible mechanism of AnxA6 translocation within the MV membrane bilayer. First, annexins in the lumen of MVs bind to phosphatidylserine (PS) of the inner leaflet of the MV bilayer in the presence of Ca^2+^ triggering the formation of a nucleational core (NC) with the aid of AnxA5 and other proteins (Figure 8A,B) [22,47]. AnxA6 may also contribute to the accumulation of Ca^2+^ and to stabilize Ca^2+^-binding to PS, promoting a favorable environment for apatite formation (Figure 8C). Next, a local pH drop during apatite formation may promote the insertion of AnxA6 into the MV bilayer (Figure 8D), which makes AnxA6 resistant to EGTA extraction [48], as confirmed in this work. Whether the transmembrane AnxA6 has the ability to transport Ca^2+^ is not yet settled, although several reports have suggested that AnxA5 [40,49,50,51] or unspecified annexins [52,53] can induce Ca^2+^ influx. The insertion of AnxA6 induced by acidic pH is reversible upon pH increase [13], which suggests that AnxA6 could be expelled in the extracellular matrix (ECM), where pH is neutral, or it could also bind to phosphatidylcholine (PC) on the MV surface, mimicking the association of AnxA6 with the outer leaflet of the MV membrane bilayer (Figure 8E). This may occur either during translocation of AnxA6 from the lumen toward the extracellular leaflet of MVs or by its presence on the surface of just released MVs to seal membrane holes. Extracellular AnxA6 may contribute to enforce MV interactions with collagen fibers (Figure 8F). The proposed mechanisms of translocation and AnxA6 localizations are consistent with the distinct types of bindings of AnxA6 to DPPS with and without Ca^2+^, as well as to DPPC with and without Ca^2+^.

## Figures and Tables

**Table 1:** Values of exclusion pressure (π*_exc_*) for lipid monolayers and concentrations of protein incorporated into lipid bilayers.

Lipid Composition (Molar Ratio)	Ca^2+^ (2mM)	π_exc_ in Monolayers (mN/m)	[Protein] Incorporated in Liposomes (µg/mL)
DPPC	−	22.2 ± 2.5	18.0 ± 0.4
	+	25.1 ± 2.7	59.0 ± 1.1
DPPC:DPPS (9:1)	−	23.7 ± 3.4	17.0 ± 0.3
	+	32.9 ± 2.0	43.0 ± 0.9
DPPC:Chol (6:4)	−	ND*	0.60 ± 0.01
	+	ND*	13.0 ± 0.3
DPPC:Chol:DPPS (5:4:1)	−	36.7 ± 1.0	19.0 ± 0.4
	+	34.3 ± 1.3	54.0 ± 1.0

*Did not form stable monolayer.

**Table 2:** Differential scanning calorimetry (DSC)-calculated thermodynamic parameters [enthalpy (ΔH), transition phase temperature (T_m_) and phase transition cooperativity (Δt_1/2_)] for liposomes (10 mg/mL) and proteoliposomes with different lipid compositions, harboring or not AnxA6, and in the presence and absence of Ca^2+^ (2 mM).

		Without Ca^2+^			With Ca^2+^		
Lipid Composition (Molar Ratio)	AnxA6	ΔH (Kcal/mol)	T_m_ (°C)	Δt_1/2_	ΔH (Kcal/mol)	T_m_ (°C)	Δt_1/2_
DPPC	−	9.25	40.8	1.00	8.08	40.8	1.02
	+	6.75	41.2	1.07	6.08	41.0	1.09
DPPC:DPPS (9:1)	−	6.56	41.6	1.42	8.72	42.3	2.80
	+	5.29	41.7	1.50	4.96	42.7	2.29
DPPC:Chol (6:4)	−	1.24	46.7	29.59	0.57	44.3	22.86
	+	0.70	46.2	26.97	0.36	49.5	24.66
DPPC:Chol:DPPS (5:4:1)	−	5.36	43.6	14.54	1.14	46.3	19.37
	+	0.06	42.4	1.63	0.43	46.2	13.89
